# Sex differences in the clinical characteristics and brain gray matter volume alterations in unmedicated patients with major depressive disorder

**DOI:** 10.1038/s41598-017-02828-4

**Published:** 2017-05-30

**Authors:** Xiao Yang, Zugui Peng, Xiaojuan Ma, Yajing Meng, Mingli Li, Jian Zhang, Xiuliu Song, Ye Liu, Huanhuan Fan, Liansheng Zhao, Wei Deng, Tao Li, Xiaohong Ma

**Affiliations:** 1 Psychiatric Laboratory and Mental Health Center, the State Key Laboratory of Biotherapy, West China Hospital of Sichuan University, Chengdu, China; 2 Huaxi Brain Research Center, West China Hospital of Sichuan University, Chengdu, China; 3 Mental Health Center, West China Hospital of Sichuan University, Chengdu, China; 4 Chengdu First People’s Hospital, Chengdu, China

## Abstract

This study was to explore the sex differences in clinical characteristics and brain gray matter volume (GMV) alterations in 29 male patients with major depressive disorder (MDDm), 53 female patients with MDD (MDDf), and in 29 male and 53 female matched healthy controls. Maps of GMV were constructed using magnetic resonance imaging data and compared between groups. We evaluated clinical symptoms using the Hamilton Rating Scale for Depression and obtained a total score and five syndrome scores. A two-factor ANCOVA model was specified using SPM8, with sex and diagnosis as the between-subject factors. We found that: (1) significant GMV increase in the left cerebellum and GMV reduction in the bilateral middle temporal gyrus and left ventral medial prefrontal gyrus occurred selectively in male patients, while the GMV reduction in the left lingual gyrus and dorsal medial prefrontal gyrus occurred selectively in female patients; (2) MDDf may have experienced more severe sleep disturbance than MDDm; and (3) the severity of sleep symptom could be predicted by the sex specific brain structural alterations in depressions. These findings suggest that sex specific anatomical alterations existed in MDD, and these alterations were associated with the clinical symptoms.

## Introduction

Sex differences are observed in epidemiological and clinical aspects of major depressive disorder (MDD). Male and female patients with depression show significant differences in some important clinical features such as morbidity, suicide rate, and clinical symptoms. Studies have consistently shown that females are diagnosed with depression twice as often as males^1, 2^. The greater prevalence of depression in females does not seem to be due either to differences in the rates of reported stressful life events or to differential sensitivity to their pathogenic effect^3^. The sex difference may be partly due to the development of the brain and its reaction to stress and neurotoxic substances^4^. Anyway, the widely accepted “female preponderance” of MDD has led to questions concerning sex effects on the cause of depression.

Besides, suicide rates for females are usually three to five times higher than that for males. Although female patients with MDD are more likely to commit suicide, male patients with MDD are more likely to be successful when they commit suicide, and thus are at a higher risk for successful suicide^5^. In addition, females with MDD typically report more symptoms than male patients, including appetite or weight changes, sleep disturbances, and feelings of worthlessness or guilt^6^. Another study had further confirmed sex differences in clinical features. In this study, 206 males and 292 females who were nonpsychotic and unipolar depressive were recruited and their symptoms were assessed. Results showed that more females with depression tended to have severe symptoms associated with depression such as increased appetite and weight gain, fatigue, and sleep disturbance^7^. Such a remarkable heterogeneity, varied clinical presentation, and a stable ratio of sex difference in MDD demonstrate that two forms of depression with different phenotypes may exist, with their neurobiological basis poorly understood.

A great interest has long been aroused in exploring the neural mechanisms underpinning the sex-related differences in depression. As a non-invasive method, magnetic resonance imaging (MRI) technologies are beginning to provide new insights into the underlying mechanisms of brain^8^. In general, neuroimaging researches have found that sex affects structural brain imaging results. In a previous neuroimaging study where male and female patients with depression were compared with their sex-matched healthy controls (HCs))^4, 9^, male patients had a significantly smaller hippocampal volume than female patients. Another study further identified that hippocampus volume tended to decrease in male patients while increase abnormally in female patients^4^. In previous studies, however, hippocampal volume was found larger in males than in females, and the decreased hippocampus volume became more significant in males than in females with aging^4, 10^. Therefore, male patients with depression may have an accelerated age-related decrease in hippocampal volume, whereas hippocampal volume alterations in female patients with depression may have a different mechanism^11^. These reminded us of a possible sex difference in the mechanism of brain abnormalities among patients with MDD.

What is more, gender also affects medication response of brain volume in patients of both sex. For instance, female responders had a larger hippocampal volume than female non-responders, but male patients did not have the same response^12, 13^. Moreover, untreated patients with depression usually have a smaller hippocampal volume than healthy controls^14^. These findings suggest that antidepressant treatment may exert a protective effect against the decrease in hippocampus volume in female responders^15^. In this sense, larger hippocampus may function as a potential protective neurobiological factor and help enhance treatment efficacy in female responders^11^ but not in male patients. All these studies indicate a possible sex difference in the mechanism of response to antidepressant treatment.

The purpose of the present study was to explore the sex differences in the phenotypes of depression including clinical symptoms, brain abnormalities as well as their relationships. We evaluated clinical symptoms of patients using the 17-item Hamilton Rating Scale for Depression (HAMD) and obtained a total score and five syndrome scores for anxiety, weight, cognitive, retardation, and sleep disturbance. Based on the existing literatures, we hypothesized that: (1) males and females with MDD may show a sex difference in some phenotypes, including the level of brain gray matter volume and clinical symptoms; and (2) their sex specific brain alterations may associate with their clinical characteristics.

## Results

### Demographic and clinical characteristics of participants

In this study, 29 MDDm and 53 MDDf patients, together with 29 male and 53 female healthy controls (HCm, HCf) who were well matched in age, education year, and intelligence quotient were studied. No significant differences were found among any of the groups in age, education year, or intelligence quotient. In addition, no significant differences were observed between male and female patients with MDD in age of onset, the mean disease duration (month), number of episode, HAMD total score, syndrome of anxiety, syndrome of weight, syndrome of cognitive disturbance, or syndrome of retardation. Compared to MDDf group, MDDm group had a lower value of syndrome of sleep disturbance (t = −1.99, p = 0.050). Sleep disturbance was assessed by the items of HAMD 4, 5, and 6. MDDf group scored higher scores in early morning awakening (HAMD 6) than MDDm group (t = −2.16, p = 0.035) while no statistically significant differences were found between the two groups in difficulty falling sleep or frequent awakenings (Table 1).

**Table 1:** Demographic and clinical characteristics of participants.

Items	MDDm (n = 29)	MDDf (n = 53)	HCm (n = 29)	HCf (n = 53)	*P*
Age	27.48 ± 7.55	30.21 ± 10.79	26.38 ± 6.91	29.06 ± 9.10	0.429
education year	14.48 ± 2.28	13.00 ± 3.11	15.17 ± 3.48	13.53 ± 3.98	0.275
IQ	109.97 ± 11.86	108.48 ± 11.79	112.29 ± 12.27	108.94 ± 15.27	0.625
Age of onset	25.448 ± 8.386	28.038 ± 10.202	—	—	0.247
Disease duration (months)	26.66 ± 43.50	33.19 ± 50.16	—	—	0.557
Number of episode	1.66 ± 1.11	1.43 ± 0.91	—	—	0.334
HAMD Total score	23.21 ± 4.14	23.00 ± 4.11	—	—	0.829
Anxiety/somatization	4.62 ± 1.35	5.13 ± 1.32	—	—	0.099
Weight	1.55 ± 0.78	1.30 ± 0.89	—	—	0.209
Cognitive disturbance	4.10 ± 1.42	3.91 ± 1.58	—	—	0.577
Retardation	8.28 ± 1.56	7.68 ± 1.98	—	—	0.165
Sleep disturbance	3.45 ± 1.38	4.06 ± 1.29	—	—	0.050
HAMD4 (difficulty falling asleep)	1.38 ± 0.62	1.53 ± 0.64	—	—	0.308
HAMD5 (frequent awakenings)	1.17 ± 0.71	1.32 ± 0.64	—	—	0.355
HAMD6 (early morning awakening)	0.90 ± 0.62	1.21 ± 0.63	—	—	0.035

MDD: Major Depressive Disorder; MDDm: male patients with major depressive disorder; MDDf: female patients with major depressive disorder. HCm: Healthy male control; HCf: Healthy female control. IQ: intelligence quotient. HAMD: Hamilton Depression Rating Scale; Anxiety/somatization: HAMD 10, 11, 12, 15, 17; Weight: HAMD 16; Cognitive disturbance: HAMD 2, 3, 9; Retardation: HAMD 1, 7, 8, 14; Sleep disturbance: HAMD 4, 5, 6.

### Gray matter volume (GMV) alterations in MDD patients

**Figure 1:**
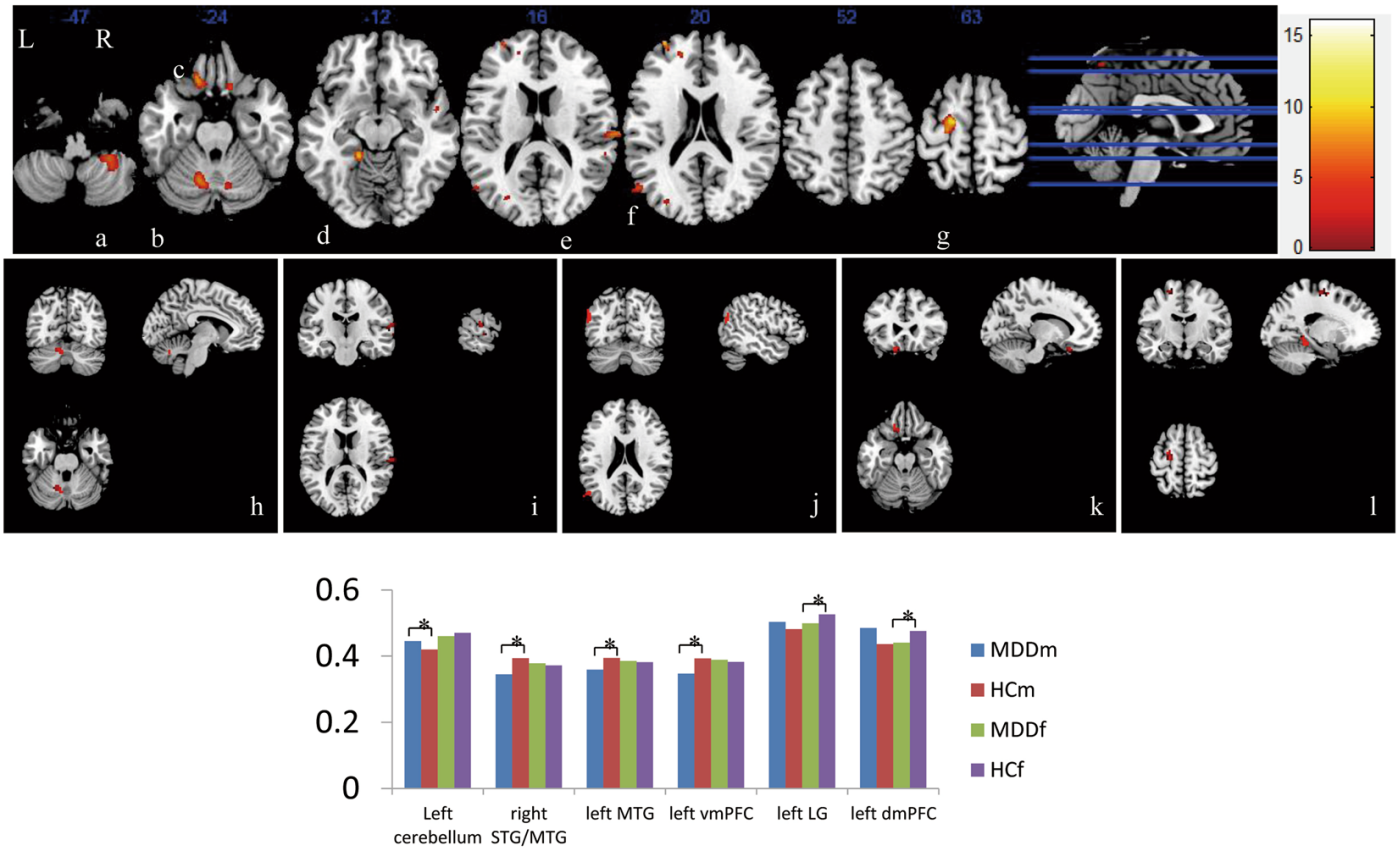
Sex by diagnosis interaction in GMV maps. Significant sex by diagnosis interaction was found in A: right cerebellum, B: left cerebellum, C: left ventral medial prefrontal gyrus (rectal gyrus), D: lingual gyrus, E: right middle/superior temporal gyrus, F: left middle temporal gyrus, and G: left dorsal medial prefrontal gyrus. Simple effect analysis suggested that male patients with MDD showed selective GMV increase in the left cerebellum (**a**) and reduction in the right superior/middle temporal gyrus (**b**), left middle temporal gyrus (**c**) and left ventral medial prefrontal gyrus (**d**), while female MDD patients showed selective GMV reduction in the left lingual gyrus (**e**) and dorsal medial prefrontal gyrus (**e**). The bar chart indicates the average GMV of regions with alterations. *Represents a significant difference detected. L, left hemisphere; R, right left hemisphere.

The regions showing significant differences between groups in the GMV are listed in Table 2. Compared with controls, patients with MDD showed decreased GMV in the right orbital gyrus, bilateral superior/middle/inferior frontal gyrus, right lingual gyrus, superior/inferior parietal gyrus, right insula, and left cerebellum. Significant sex by diagnosis interactions was found in the bilateral cerebellum, middle temporal gyrus, left ventral medial prefrontal gyrus (vmPFC), left dorsal medial prefrontal gyrus (dmPFC), and left lingual gyrus. Post hoc pair-wise comparisons showed that the significant GMV increase in the left cerebellum and reduction in the right superior/middle temporal gyrus (STG/MTG), left middle temporal gyrus (MTG) and vmPFC occurred selectively in male patients, while the GMV reduction in the left lingual gyrus (LG) and dmPFC occurred selectively in female patients (Table 2) (Fig. 1). With regard to the main effect of sex, compared to females, males had increased GMV in the bilateral posterior cingulate gyrus extending to precuneus, bilateral lingual gyrus extending to cuneus, left inferior occipital gyrus and left orbital frontal gyrus, but had deceased GMV in the right hippocampus extending to temporal gyrus and thalamus, bilateral caudate, bilateral insula, left superior/middle temporal gyrus, bilateral parietal gyrus, bilateral superior orbital frontal gyrus, bilateral rectal gyrus and bilateral cerebellum.

**Table 2:** Sex and diagnosis effects on voxel-wise GMV characteristics.

Comparisons	Regions	Voxels	t/F Score	X, Y, Z	L/R	BA
**Interaction of sex and diagnosis**						
MDDm > HCm	Cerebellum	259	12.49	−14, −60, −26	L	—
MDDm < HCm	Middle/superior temporal gyrus	323	13.52	67, −19, 15;	R	40
MDDm < HCm	Middle temporal gyrus	286	10.54	−54, −67, 19;	L	39
MDDm < HCm	Ventral medial prefrontal gyrus (rectal gyrus)	313	13.27	−15, 26, −26	L	47
MDDf < HCf	Lingual extending to parahippocampa gyrus	236	14.91	−17, −42, −9	L	19/30
MDDf < HCf	Dorsal medial prefrontal gyrus extending to supplementary motor area (SMA)	349	16.11	−18, −9, 61	L	6
	Cerebellum	407	11.09	29, −42, −47	R	—
**Main effect of sex**						
Male > Female	Posterior cingulate gyrus extending to precuneus	933	102.33	−2, −48, 15	L/R	29/30
	Lingual gyrus extending to cuneus	3631	61.75	3, −96, 6/−6	L/R	17/18
	Middle occipital gyrus	291	28.48	−42, −78, 10	L	10
	Occipital gyrus extending to cerebellum posterior lobe	649	28.18	−53, −73, −21; −43, −84, −21	L	18/19
	Inferior orbital frontal gyrus	209	21.12	−32, 48, −18	L	11
Male < Female	Hippocampus extending to temporal gyrus, thalamus, caudate	4064	30.75	27, −31, −6; 57, −28, 3	R	21/22/27
	Caudate extending to putamen	3307	48.92	−6, 8, 3; 8, 12, 1	L/R	—
	Insula	1380	26.84	42, 14, −8	R	47
	Insula	772	20.63	−35, 13, −6	L	13/47
	Superior/middle temporal gyrus	2561	26.24	−50, −40, 7	L	22
	Superior/middle temporal gyrus	136	11.12	−57, 3, −11	L	21/38
	Parietal gyrus extending to frontal gyrus	7219	25.40	6, −33, 76; 36, −24, 49	L/R	3/4/6
	Superior orbital frontal gyrus	955	21.37	10, 67, 3; −9, 69, 3	L/R	10
	Rectal gyrus	653	12.61	−11, 33, −21; 5, 36, −25	L/R	11
	Postcentral gyrus	153	11.47	−53, −7, 34	L	6
	Cerebellum extending to hippocampus	35953	48.18	5, −70, −27; −9, −78, −53	L/R	—
**Main effect of diagnosis**						
MDD < HC	Orbital gyrus	1247	26.73	47, 53, −8; 35, 51, 4	R	10
	Superior/middle frontal gyrus	6842	25.69	−30, 54, 21; −17, 64, 20	L	9/10
	Middle/inferior frontal gyrus	488	17.67	46, 10, 30	R	9
	Superior/middle frontal gyrus	1013	17.16	30, −9, 63	R	6/8
	Lingual gyrus extending to parahippocampa gyrus	594	16.62	23, −75, −8	R	18/19
	Middle/inferior frontal gyrus	546	16.13	−46, 0, 33;	L	6/9
	Insula	390	15.10	47, −1, −6	R	22
	Superior/inferior parietal gyrus	196	14.82	47, −39, 57	R	40
	Cerebellum	149	11.64	−36, −51, −24	L	—

All clusters were identified using the threshold of *p* < 0.005 AlphaSim corrected (i.e., *p* < 0.005 combined with a minimal cluster size of 132 voxels). All clusters were identified using post hoc two-sample t-tests within masks from *F*-contrasts (interaction or main effects). GMV: gray matter volume. L, left; R, right; BA, Brodman area.

### Prediction of the severity of sleep symptom in depressions

The mean GMV values of the bilateral cerebellum, MTG, left vmPFC, left dmPFC, and left LG were extracted and correlated with clinical characteristics. We found that the increase of GMV value in left cerebellum could predict the severity of sleep symptom in male patients with MDD (*β* = −0.372, *p* = 0.047, uncorrected), and that the decrease of GMV value of left LG could predict the severity of sleep symptom in female patients with MDD (*β* = −0.302, *p* = 0.028, uncorrected) (Table 3). No other significant correlations were detected.

**Table 3:** Predictors of sleep disturbance in patients with MDD.

Dependent variable	Independent variable	*B* score	*β* score	*T* score	*p* value
**MDDm**					
symptom of sleep	Constant	−2.223	—	−0.812	0.424
	GMV of left cerebellum	12.721	0.372	2.080	0.047
**MDDf**					
symptom of sleep	Constant	7.974	—	4.575	0.000
	GMV of left lingual gyrus	−7.841	−0.302	−2.258	0.028

MDD: Major Depressive Disorder; MDDm: male patients with major depressive disorder; MDDf: female patients with major depressive disorder. GMV: gray matter volume.

## Discussion

The present study is one of the first large clinical studies in which sex difference in characteristics of MDD patients are examined by combining clinical presentation and neuroimaging phenotypes, using well-established measures.

We found that the sex by diagnosis interactions were found in the bilateral cerebellum, MTG, left vmPFC, left dmPFC, and left LG. Post hoc pair-wise comparisons showed that the significant GMV increase in the left cerebellum and reduction in the bilateral MTG and left vmPFC occurred selectively in male patients, while the GMV reduction in the left LG and dmPFC occurred selectively in female patients.

Almost all previous studies on depression reported significant decrease of GMV in the prefrontal gyrus (PFC) regions although many confounding factors were not excluded^16^. Dysfunction of PFC was involved in some depressive syndromes, such as impaired attention, psychomotor retardation and executive dysfunction^17^ and associated with depression treatment^18, 19^. Left-sided PFC hypofunction appears to be predominantly associated with depressive conditions. Its activation, which has been conceptualized as preventing small stressors from becoming big stressors, may be especially relevant to the left PFC deficits seen in depressions, where small stressors indeed become overwhelming^20^. Recently, a meta-analysis also identified decreased GMV in PFC in depressions^21^. Abnormalities in the function, structure^22^, and brain circuit related to this region^23^ were all reported to be involved in emotion regulation^24^. These studies suggest that the PFC may be the key area of brain damage and disease treatment in both male and female patients with depression.

Nevertheless, our study results indicate that the reduction of vmPFC and dmPFC occurred in male and female patients, respectively. In recent years, the medial PFC (mPFC) has aroused increasing attention for its role in depression^23^. Previous neuroimaging studies have found that abnormal dmPFC activity has been linked to altered self-reflection and rumination in depression^25^. Compared to men, women are more likely to attend to and ruminate about their emotions. For women high in depression, greater rumination is associated with greater depressive symptoms^26^. The complex nature of this association may partially explain why females showed significant GMV alteration of dmPFC in our current study. The dmPFC has also been implicated in emotion-regulation processes, particularly in the down-regulation of negative affect^27^. Thus, given that females tend to recruit more PFC regions during emotion regulation, volume alteration in the dmPFC may be particularly obvious for females, leading to a dysregulation of negative affect and increased depressive symptoms. Recent research on patients with selective dorsal PFC (highest lesion overlap in the dmPFC) and vmPFC lesions even revealed that vmPFC lesions were linked with stronger resistance to depression, whereas lesions on the dmPFC were associated with vulnerability to depression^28^. This finding also goes in line with the higher prevalence of depression in females. Based on our study results and in conjunction with evidence from previous researches, we conclude that lower dmPFC GMV may serve as a vulnerability mechanism for the development of depression. Future studies are necessary to verify this possibility by examining dmPFC GMV in relation to other known risk factors for depression.

With regard to other sex differences in GMV of other brain regions, our study found that only MDDm patients showed significantly increased GMV in left cerebellum and reduction GMV in temporal gyrus while MDDf patients had significantly decreased GMV in left LG extending to parahippocampal gyrus. Since Schmahmann and Sherman first highlighted the possible important role of cerebellum in emotion regulation in 1998, cerebellum has attracted wide attention among researchers^29^. A previous study also confirmed that increased volume of cerebellum only existed in male patients with MDD^30^. One explanation is that increased GMV may be related to preapoptotic osmotic changes or hypertrophy, marking areas of early neuronal pathology^31, 32^. The larger volume of cerebellum might arise from abnormalities in connectivity or as compensatory responses to the PFC dysfunction in male depressions rather than in female depressions.

Moreover, the sex difference of brain alterations in depressions may explanation inconsistent results of previous studies. These inconsistencies might be attributed to the heterogeneity of MDD patients. Controversial structural abnormalities of insula and thalamus in MDD patients have also been reported in previous studies^33, 34^. Kong *et al*. found GMV of the insula increased in medication-naïve MDD patients^33^ while a reduction of GMV in the insular cortex was observed in medication-naïve MDD patients in another study conducted by Lai *et al*.^35^. The contradictory results regarding insula in these studies very possibly resulted from the mixture of the patients without differentiating male and female instead of from the number of episode or other factors such as medication treatment. This also called our attention to the effect of sex difference on brain alterations of MDD. Parahippocampal gyrus is a key region of the limbic system that plays a central role in the regulation of emotion, memory, motivation, and affective dimension of pain^36, 37^. This region is extensively connected with other cortical and subcortical regions in the frontal and temporal lobes and plays an important role in cognitive processes of MDD^23^. Thus, the difference of gray matter damage in parahippocampal gyrus may contribute to differences in epidemiological and clinical manifestation of depression. Although the specific mechanism underlying different brain alterations in male and female patients remains unclear, the present study still reminds us of the sex difference in patients.

Insomnia is a particularly frequent complaint which is reported by more than 90% of depressed patients^38^, and it is even more common in female^6, 7^. In the current study, we confirmed that female patients with MDD may have experienced more severe sleep disturbance, waking up early in particular, than male patients with MDD. This finding needs to be taken into account when balancing the pros and cons of treatment with drugs that can cause insomnia, especially when treating female known with insomnia risk factors. Sleep disturbance is a significant risk factor for the onset, exacerbation, and relapse of mood disorders^39^ and an increased risk of suicide^40^. Even if a female with depression is not having sleep disturbance at present, we still need to keep an eye on her sleep quality to detect and resolve problems early and timely.

Apart from sex differences in clinical features and brain abnormalities, we also identified sex specific brain alterations related to sleep disturbance in depression. We found that the increase of GMV value in left cerebellum could predict the severity of sleep symptom in male depressions, and the decrease of GMV value of left LG could predict the severity of sleep symptom in female depressions. Although the results were uncorrected, they can indicate a trend. Further studies are warranted to verify the findings.

During Rapid Eye Movement sleep, the cerebellum has been hypothesized to regulate autonomic inputs from the amygdala, periaqueductal gray, and thalamus and to express parasympathetic and sympathetic outputs to the brainstem ventilatory and oculomotor neurons^41^. Moreover, the cerebellum, to form a feed-forward loop through the thalamus and to form a feedback loop through the pons, interconnects a network with extensive cortical and subcortical areas^42^. During Non-rapid Eye Movement sleep, the thalamus receives afferents from the cerebellum changes produced after electrical stimulation or suppression of various cerebellar nuclei^43^. A recent study reported that the functional connectivity between cerebellum and frontal cortex, thalamus, precuneus, partial and temporal was impaired in sleep deprivation compared to normal sleep^44^. A growing number of evidence suggests that our visual system continues to be plastic during sleep. A more recent study explored the relation between the alterations of visual cortex and sleep, and found that increasing sleep depth is accompanied by an increasing rightward asymmetry of regions in visual cortex including the right LG^45^. Nevertheless, the specific role that cerebellum or LG plays in the regulation of sleep still remains largely unclear; this current study study just reminds us that the differences of sleep disturbance between male and female patients with depression may rely on a biological basis. In the present study, we sought to identify more objective biomarkers than chief complaints only to predict and evaluate the symptoms of sleep problems in patients with depression.

Over all, the present study found sex differences in some phenotypes of MDD; and these differences involved clinical manifestation and brain structure, and the association between sleep disturbance and brain abnormalities. Females having depression usually experience more severe sleep disturbance and have alterations in dmPFC and LG. The increase of GMV value in left cerebellum could predict the severity of sleep symptoms in male depressions, and the decrease of GMV value of left LG could predict the severity of sleep symptoms in female depressions.

Despite the findings, however, our study has three limitations. First, we did not separate the first-episode patients from recurrent patients. This limitation made us unable to see whether these abnormalities are more severe or otherwise different in patients before antidepressant treatment, or to exclude interference of the previous treatment. Second, we did not follow up the patients in our study. This made us unable to determine whether the treatment efficacy was different between male and female patients with depression. Finally, no objective measures of sleep disturbance, such as polysomnography and actigraphy, were performed in this study. Relying on the scales alone may not fully capture the presence of symptoms. In conclusion, our results suggest sex specific anatomical alterations in MDD, and such alterations were associated with clinical symptoms.

## Methods

### Participants

Eighty-two drug-free MDD patients (male/female: 29/53; mean age: 27.48 ± 7.55/30.21 ± 10.79 year) were recruited from the outpatient clinic and in-patient facilities at the Department of Psychiatry, West China Hospital of Sichuan University.

All patients met the criteria for major depressive disorder according to the Structured Clinical Interview of DSM-IV-TR (SCID-I/P) criteria as diagnosed by two professional psychiatrists (Prof. Ma XH and Prof. DW). No patients with MDD had current comorbid Axis I diagnosis. The clinical symptoms of patients were evaluated using the 17-item Hamilton Rating Scale for Depression (HAMD), which provided a total score and five syndrome scores for anxiety, weight, cognitive, retardation, and sleep disturbance. All patients were having a major depressive episode, with their HAMD 17-item scores being at least 17 on the day of scanning. Patients who had taken any antidepressants during the past 3 months before scanning were excluded. In addition, exclusion criteria included age younger than 18 years or older than 55 years, pregnancy, neurological or internal systemic diseases, a history of acute physical illness, a history of head injury resulting in loss of consciousness, and a major neurological disorders, cardiovascular disease, mental retardation, substance abuse or dependence, and general contraindications for MRI. The final sample consisted of 82 patients and 82 matched controls. At the time of the study, 60 patients were drug-naive and 22 had been medication-free for at least 3 months.

Eighty-two age, education year, and intelligence quotient matched healthy controls were recruited via advertisements within the local community (Table 1). Each participant was also interviewed by the same professional psychiatrists (Prof. Ma XH and Prof. DW) using the Structured Clinical Interview for DSM-IV, non-patient edition (SCID-I/NP), to assure that none of them had a current or past history of depression or other axis I disorders or with a history of psychiatric illness in their first-degree relatives.

All participants were 18–55 years old, right-handed Han Chinese, and provided written, informed consent. Ethical approval was obtained from the Ethics Committee of Sichuan University. All the study procedures were carried out according to the Helsinki Declaration.

### MRI scan acquisitions

The imaging data were acquired using a 3-Tesla whole body MR scanner (Achieva, Philips, Netherlands) with an eight-channel phased-array head coil. High-resolution T1-weighted images were obtained using a 3-dimensional, sagittal, magnetization-prepared rapid gradient echo (MPRAGE) sequence with the following parameters: repetition time = 8.4 ms; echo time = 3.8 ms; flip angle: 7°; in-plane matrix resolution = 256 × 256; field of view = 256 × 256 mm; thickness = 1 mm; and number of slices = 188. During scanning, all participants were ear-plugged and foam-padded, and were instructed through headphones to remain motionless.

Two experienced radiologists inspected the raw image data qualitatively. No gross abnormalities were observed for any participant.

### Data preprocessing

These T1-weighted images were analyzed using SPM8 (http://www.filion.ucl.ac.uk/spm/software/spm8/) and voxel-based morphometry (VBM8) toolbox implemented with the MATLAB toolbox (MathWorks, Inc., Natick, MA, USA). First, all T1-weighted images were realigned manually according to the anterior and posterior commissure (AC-PC) line and midsagittal plane. Second, all the T1-weighted images were segmented into probability maps of gray matter (GM), white matter (WM) and cerebrospinal fluid (CSF) using the ‘new segment’ routine implemented in SPM8. Finally, the gray matter images were spatially normalized to MNI space and smoothed with 8 mm full width at half maximum Gaussian kernel.

### Statistical Analysis

Student’s t -test and analysis of variance (ANOVA), as appropriate, were used to compare the demographic and clinical data between the subgroups. The software package utilized for this analysis was Statistical Package for the Social Sciences (SPSS 17.0 for Windows). A two-factor ANCOVA model was specified using SPM8, with sex (male, female) and diagnosis (MDD, HC) as the between-subject factors, and age, gray matter volume, and whole brain volume were included as covariates. The sex by diagnosis interaction, and the main effects of sex and diagnosis were tested. When interaction effects occurred, post hoc pair-wise comparisons were performed using two-sample t -tests within the interaction masks.

For all voxel-wise comparisons, a combination threshold of p < 0.005 for each cluster size >132 voxels was considered significant, which corresponded with a corrected p < 0.05 (using Monte Carlo simulation with the parameters including: 1000 simulations, full width at half maximum = 8 mm, cluster connections radius = 5 mm) (http://afni.nimh.nih.gov/pub/dist/doc/manual/AlphaSim.pdf).

For all regions showing sex-specific brain alteration, the correlations of the mean GMV values in these regions with subscale score of sleep disturbance were determined to investigate the clinical correlates of the brain abnormality patterns in male and female patient groups, respectively.
